# CT-guided percutaneous microwave ablation combined with bone cement injection for the treatment of transverse metastases: A case report

**DOI:** 10.1515/med-2023-0753

**Published:** 2023-07-25

**Authors:** Hongtao Hu, Lei Xu, Xiang Guo, Haijun Teng, Wenhua Liu

**Affiliations:** Department of Orthopedic Surgery, The Affiliated Hospital of Weifang Medical College, Weifang 261000, Shandong, China

**Keywords:** metastatic tumor, microwave ablation, bone cement, transverse process

## Abstract

Metastatic diseases of the spine are becoming increasingly common with an aging population and improvements in systemic cancer therapies. Microwave and vertebroplasty are the mainstay modalities for treating painful spine metastases. Most early spinal metastases predominantly attack the adnexa, but there are few reports on its treatment. This report presents a case of a 56-year-old female who had experienced severe thoracic back pain for several days and was diagnosed with a metastatic tumor of the right transverse process of T7. Percutaneous microwave ablation in combination with bone cement injection was used to treat the metastatic tumor under CT guidance. The postoperative pain on the Visual Analogue Scale was 1/10, without nerve or vessel damage and bone cement leakage during the operation.

## 1 Introduction

Spinal metastases are most common in bone metastasis, which is observed in 60–70% of patients with systemic cancer [1]. Invasion of the vertebral body and appendix by tumor tissue is frequent, which can lead to spinal cord compression, intractable pain, pathological fractures, and other problems [2]. However, most early spinal metastases predominantly attack the adnexa in clinical practice and there are few reports on the treatment of early adnexal metastases in the spine.

Pain alleviation and spinal stability are the main targets of treatment for spinal metastases [3,4]. Percutaneous vertebroplasty, which also enables percutaneous biopsy, has been demonstrated to be an economical and effective technique for reducing pain (usually in 74–100% of patients) and avoiding additional vertebral collapse in spinal metastases [5,6]. In recent years, image-guided percutaneous ablation techniques including radiofrequency ablation and microwave ablation (MWA) have emerged as a viable therapeutic choice [7,8,9]. It can be carried out either alone or in conjunction with bone cement injection [10].

In this report, the author presents the use of MWA in combination with bone cement injection to percutaneously treat a case of early spinal metastases located on the transverse process.

## 2 Case presentation

**Figure 1:**
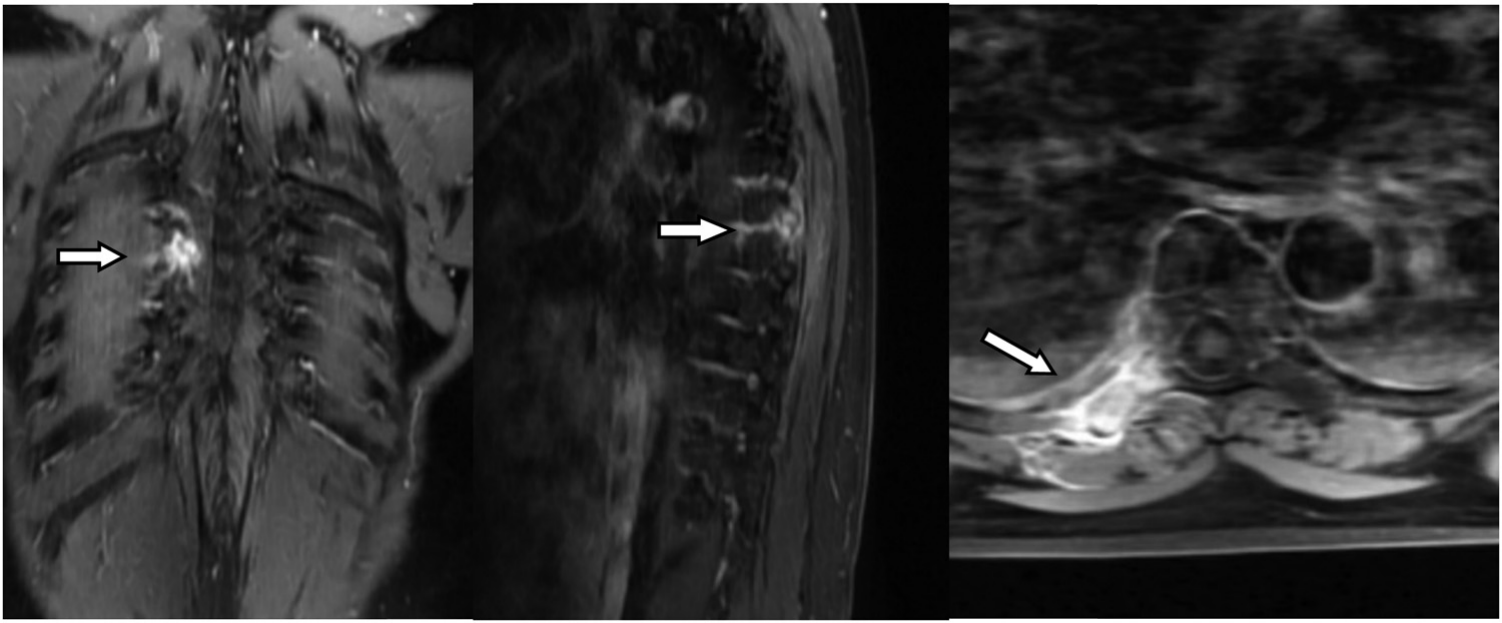
Metastatic lung cancer of the right T7 transverse process (white arrows) showing enhancement after injection of gadolinium chelates.

**Figure 2:**
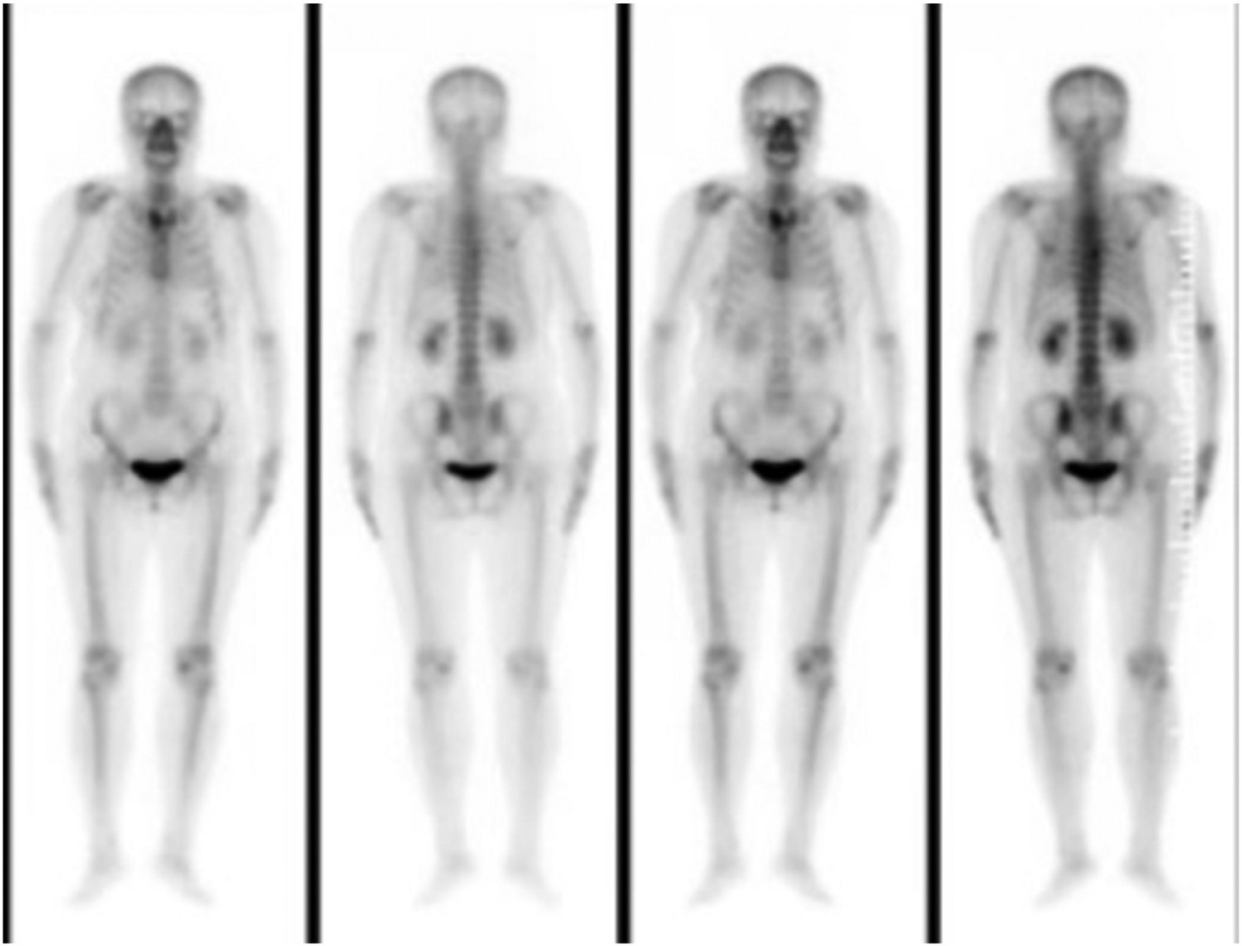
Bone scan showed diffuse increased uptake of the isotope at the level of the right transverse process of T7.

A 56-year-old female, who had lung cancer and undergoing wedge resection of the upper left lung, presented with subacute onset, progressive, right thoracic back pain for several days. Her pain worsened at night and the Visual Analogue Scale (VAS) was 8/10. A neurological exam showed normal motor function, normal sensation, and no abnormal reflexes. The patient had point tenderness over the T7 regions. Magnetic resonance imaging showed that the lesion was located at the right transverse process of T7. The vertebral body was not destroyed and the cervical spinal cord was not compressed (Figure 1). Bone scan showed diffuse increased uptake of the isotope at the level of the right transverse process of T7, without other abnormalities elsewhere in the skeleton (Figure 2).

**Figure 3:**
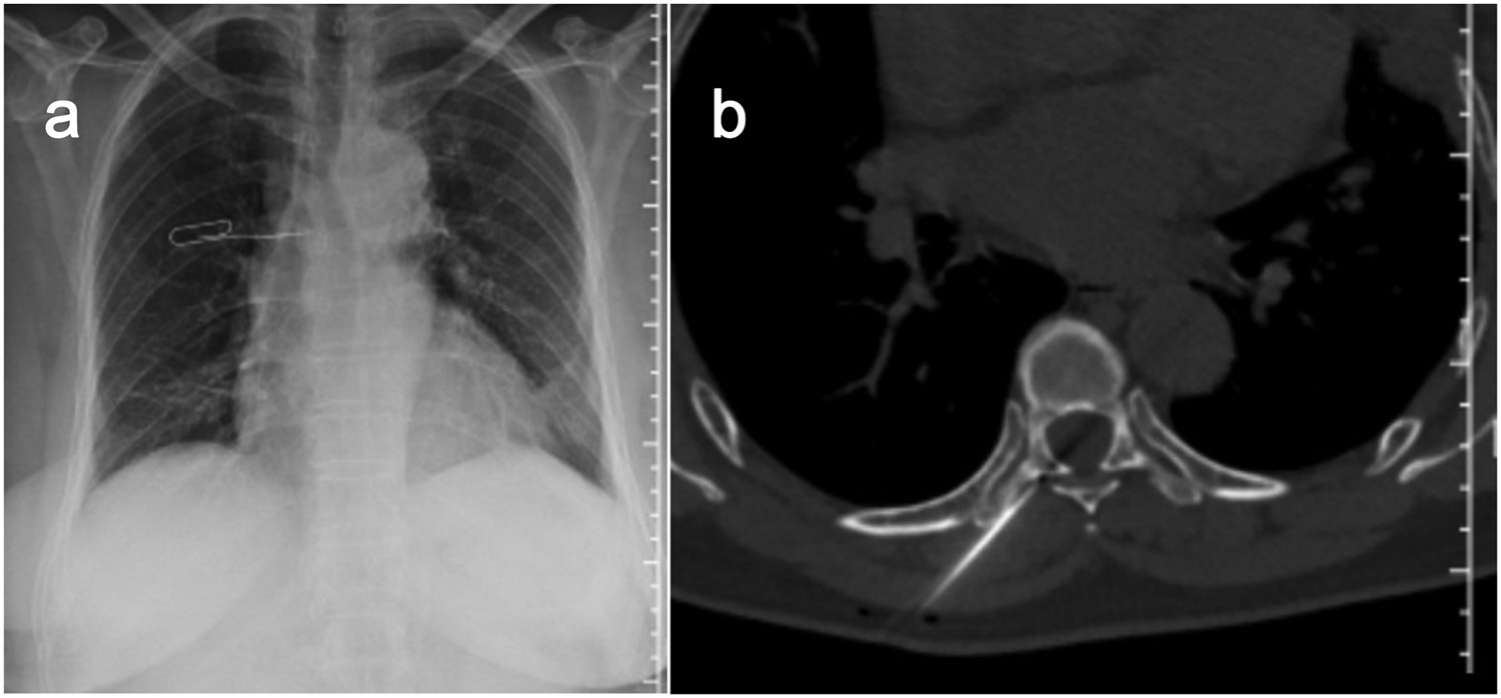
Localization is performed on the body surface by X-ray (a) and puncture is performed under CT guidance (b).

**Figure 4:**
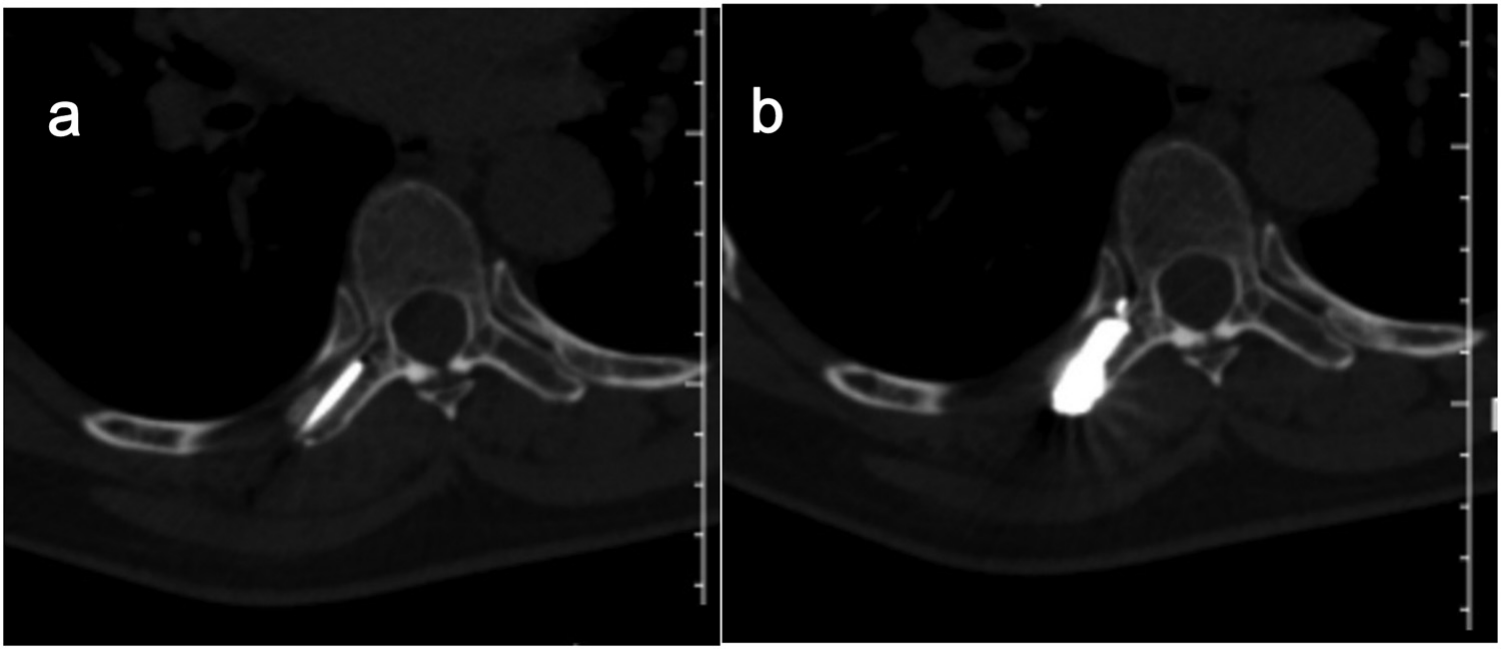
On control CT scan performed during MWA (a) and bone cement was injected into the lesion during the operation (b).

All procedures were performed using a Siemens Sensation 64 CT-scan (Siemens Healthcare, Erlangen, Germany) in the prone or supine position depending on the position of the lesion. The target lesion and needle path were located by non-enhanced helical CT acquisitions. Preoperative invasion range and destruction degree of adnexal tumor were determined and the injection route was planned according to X-ray, CT-scan, and MR (including enhanced) image data. After accurate marking of the skin and in strictly aseptic conditions, local subcutaneous injection of lidocaine 1% was performed at the defined skin entry point and an 11-gauge 15 cm co-axial needle (Dragon Crown Medical Co., LTD, China) was introduced step by step under CT-fluoroscopy (Figure 3a and b). After the needle was punctured into the right transverse process of T7 (adjusted during CT guidance), a 16G-gauge 20 cm MWA probe (ECO-100AL6, Nanjing Yigao Medical Technology Co., LTD, China) was advanced into the osseous lesions of T7’s right transverse process. Then connect the ablation needle to the ablation instrument (ECO-100A1, Nanjing Yigao Medical Technology Co., LTD, China), adjust the power to 70 W, and ablate twice for 2 min each time (Figure 4a). During the ablation process, the patient’s vital signs and the movement of the lower limbs were observed. After the ablation needle was withdrawn, the polymethylmethacrylate bone cement (PMMA, Heraeus Medical Ltd, German) was prepared in the appropriate ratio (powder (g)/liquid (mL) = 2:1) and was pushed with a cement-filled cannula (Dragon Crown Medical Co., LTD, China). The distribution of the bone cement is observed until it has diffused to the edge of the lesion (Figure 4b). After the bone cement has been filled, the cannulas were removed and the patient is returned to the ward for recovery.

**Figure 5:**
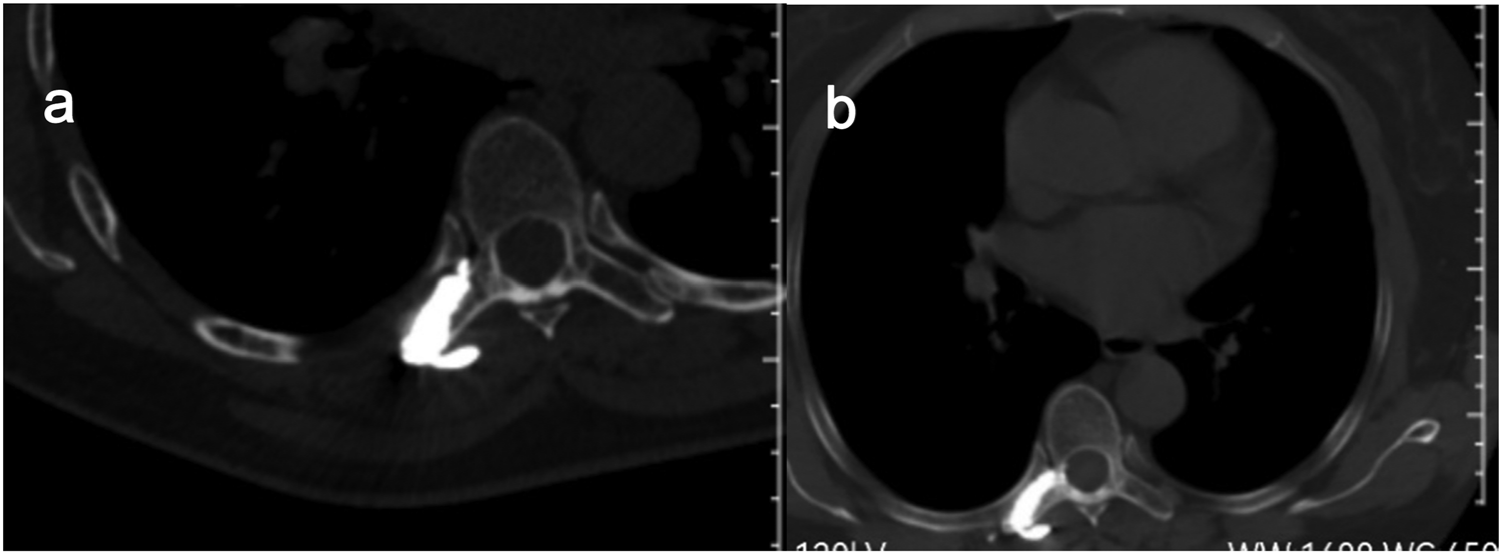
Postoperative CT showed intact cement filling and no leakage in the spinal canal (a) and follow-up after 6 months showed no displacement of the bone cement and no new bone destruction (b).


After the procedure, the patient felt painless and the VAS score was 1/10. No complications occurred during the operation. CT showed that the cement was well filled and no leaks at the right transverse process of T7 (Figure 5a). At the 6-month follow-up, the patient’s chest and back pain was relieved, and a repeat CT revealed no leakage of bone cement and no new destruction of bone (Figure 5b).

**Ethical approval:** All procedures performed in the studies involving human participants were in accordance with the ethical standards of the institutional and/or national research committee(s) and with the Helsinki Declaration. The Institutional Review Board of The Affiliated Hospital of Weifang Medical College approved the study protocol (approval document of biomedical ethics review committee of The Affiliated Hospital of Weifang Medical College, wyfy-2023-ky-146).
**Informed consent:** Written informed consent for publication was obtained from the patient.

## 3 Discussion

The survival time for patients with tumors has been vastly expanded due to advancements in technology for tumor treatment. Improving the quality of life is a clinical challenge for metastatic disease [4,11]. Palliative care is the major strategy for treating spinal metastases since it reduces suffering and enhances the quality of life. Medical treatment, surgical resection and reconstruction, local radiation, and local interventional treatment are examples of the available treatment modalities [12,13,14].

A better option for treating spinal metastatic tumors has emerged with the development of minimally invasive therapy. The primary technique of treatment for spinal metastases is vertebroplasty [6,15]. Bone cement is injected to restore the stability of the diseased vertebral body following percutaneous puncture of the puncture needle into the diseased vertebral body under fluoroscopic monitoring. The heat and self-toxicity generated during the hardening of the bone cement have the effect of killing tumor cells. The patient’s function improves and their pain is lessened after treatment [6]. However, there is a risk that the bone cement will leak during the injection and will not be able to completely enclose the tumor tissue, failing to obtain the intended results [16]. Electromagnetic waves include microwave, radiofrequency ablation, and laser interstitial thermal therapy [17]. By inserting the MWA needle into the patient’s tumor, the microwave electromagnetic radiation field’s heat induces an increase in tissue temperature, causes coagulation-type necrosis of tumor cells, inhibits tumor cell growth, and lessens the damage that tumor cells cause to the vertebral body, thereby reducing the patient’s pain and discomfort [8,9,10,18]. However, the strength of the bone cannot be increased by this treatment, and the spine’s stability is not significantly improved [19].

Spinal adnexal metastases cause microfractures within the adnexa causing irritation and injury to nerves inside and outside the adnexa, which is the most important factor in producing thoracolumbar back pain [20]. Direct invasion of nerve endings by the tumor tissue to damage and compress them is also a common cause of pain. In this article, we used MWA combined with bone cement for the treatment of transverse metastases. After the operation, the thoracolumbar back pain of the patient was rapidly relieved. In addition, MWA has a thermocoagulation effect, which can rapidly cause tumor coagulation and necrosis, vaporization, and facilitate the diffusion of bone cement to the marginal normal bone. It can play a thermocoagulation effect on the circumferential tumor. In addition, bone cement alone is cytotoxic and can cause necrosis of the surrounding tumor cells. The bone cement has to stabilize and support the effects on the vertebral attachments, and after injecting into the diseased vertebral attachments, it will fix the microscopic bone in a short period, block the loss of support of the diseased vertebral attachments due to the invasion of tumor cells [21], and reduce the stimulation of nerve roots and sinus vertebral nerves due to the instability of the spine [10,22,23]. Both of these factors can prevent further destruction of the spinal attachment and delay the infiltration of tumor cells into the vertebral body or vertebral canal while rapidly relieving the pain in the thoracic and lumbar back of patients.

Due to the spinal adnexa’s anatomical location, a CT-guided puncture is advised to guarantee the precision of the microwave needle placement. The major benefit of a CT scan is its ability to visualize the cross-section and pinpoint the target lesion with accuracy, guaranteeing that the ablation needle is inserted in the metastases’ center. To avoid the risk of intraoperative spinal cord and nerve injury, we shortened the ablation time and increased the ablation cycle with an RF power of 30 W. The ablation time was adjusted according to the location of the metastases. Two ablations of 2 min each are recommended for tumors located in the transverse process, spinous process, and articular process, and four ablations of 1 min each are recommended for tumors located in the vertebral plate and arch. In this article, the transverse process was ablated twice for a total of 2 min to lower the possibility of the intraoperative spinal cord and nerve damage. To prevent thermal injury to the surrounding normal tissues during the work of the ablation needle, it is crucial to verify that the water-cooling cycle is functioning properly before starting the microwave power output. Compared to the vertebral body, the vertebral adnexa which is smaller and situated closer to the spinal cord and nerve roots is vulnerable to bone cement leakage. In the process of making bone cement, we strictly follow the fixed ratio (powder (g)/liquid (mL) = 2:1) to ensure that the bone cement is in the “toothpaste apparatus” during the pushing process. Each injection should be between 0.3 and 0.5 mL and push injection process must be slow. To prevent bone cement from leaking, the needle’s tip should be placed away from the cortex of the damaged bone. In addition, careful observation and study of the preoperative imaging data are also essential to prevent leakage.

## 4 Limitations

This study has limitations, mostly intrinsic to the nature of case reports or case series. The poor financial situation of the patients and the short follow-up period, lack of a control group, and the single assessment index may not fully reflect the symptomatic improvement of the procedure. Future studies may need to include more cases and use better evaluation indicators.

## 5 Conclusion

In conclusion, percutaneous MWA in combination with bone cement injection can enhance spinal stability, alleviate the pain of patients with spinal metastases, and maintain better long-term efficacy than single vertebroplasty treatment, which is worthy of clinical reference.
